# Supplementary material for: Real-Time Monitoring of the Atrazine Degradation by Liquid Chromatography and High-Resolution Mass Spectrometry: Effect of Fenton Process and Ultrasound Treatment
Source: Molecules. 2022 Dec 17;27(24):9021. doi: 10.3390/molecules27249021 (PMC9785566; doi:10.3390/molecules27249021)
Supplement: Supplementary file 1 [file molecules-27-09021-s001.zip › molecules-2028557-supplementary.pdf]

Real-Time Monitoring of the Atrazine Degradation by Liquid Chromatography and High-Resolution Mass Spectrometry: Effect of Fenton Process and Ultrasound Treatment

Junting Hong [1,2], Nadia Boussetta [2], Gérald Enderlin [2], Nabil Grimi [2] and Franck Merlier [1,*]

[1] Université de Technologie de Compiègne, UPJV, CNRS, Enzyme and Cell Engineering, Centre de Recherche Royallieu, CEDEX CS 60319, 60203 Compiègne, France
[2] Université de Technologie de Compiègne, ESCOM, TIMR (Integrated Transformations of Renewable Matter), Centre de Recherche Royallieu, CEDEX CS 60319, 60203 Compiègne, France
* Correspondence: franck.merlier@utc.fr; Tel.: +33-(0)-3-44-23-73-55

Table S1. Metabolite identification of Fenton oxidation (2 eq. Fenton reagents) and ultrasound treatment (US 50 kHz + Fe^{2+}) after 50 min.

Molecular formula	Abbreviation	Name	m/z	rt (min)	C/C0(ATZ)_ Color marking: greater than 0.5 %	
					2 eq. Fenton reagents	US 50 kHz + Fe^{2+}
$C_8H_9D_5ClN_5$	ATZ-D5	Atrazine-D5	221.1324	2.58	IS	
$C_8H_{14}ClN_5$	ATZ	Atrazine	216.1010	2.56	72.842 %	76.801 %
$C_8H_{12}ClN_5O$	CDIT	Atrazine amide	230.0804	2.09	4.608 %	0.127 %
$C_8H_{14}ClN_5O_2$	HAHT	2-({4-chloro-6-[(1-hydroxyethyl)amino]-1,3,5-triazin-2-yl}amino)propan-2-ol	248.0909	2.21	4.479 %	
$C_5H_8ClN_5$	DIA	Deisopropylatrazine	174.0541	1.62	2.117 %	3.649 %
$C_8H_{12}ClN_5$	CVIT	6-chloro-N2-ethenyl-N4-(propan-2-yl)-1,3,5-triazine-2,4-diamine	214.0854	2.07	1.899 %	
$C_8H_{14}ClN_5O$	CNIT	1-({4-chloro-6-[(propan-2-yl)amino]-1,3,5-triazin-2-yl}amino)ethan-1-ol	232.0960	1.87	1.562 %	
$C_8H_{13}N_5O_2$	ODIT	Hydroxyatrazine amide	212.1142	1.21	1.072 %	0.892 %
$C_7H_{10}ClN_5O$	CDET	Simazine amide	216.0647	1.82	0.641 %	
$C_6H_{10}ClN_5$	DEA	Deethylatrazine	188.0698	1.91	0.247 %	3.073 %
$C_5H_6ClN_5O$	CDAT	Deisopropylatrazine amide	188.0334	1.26	0.047 %	
$C_7H_{11}N_5O_2$	ODET	N-[6-(Ethylamino)-4-oxo-1,4-dihydro-1,3,5-triazin-2-yl]acetamide	198.0986	1.08	0.037 %	0.327 %
$C_3H_5N_5O$	AM	Ammeline	128.0567	0.49		
$C_3H_4ClN_5$	DDA	Didealkylatrazine	146.0228	1.02		
$C_3H_5N_3O_4$	CBOI	1-carboxybiuret	148.0353	0.42		
$C_6H_{11}N_5O$	DEHA	Deethylhydroxyatrazine	170.1037	0.81		0.097 %
$C_8H_{15}N_5O$	HA	Hydroxyatrazine	198.1350	1.23		0.343 %
$C_7H_9N_5O_3$	ODDT	N,N'-(6-hydroxy-1,3,5-triazine-2,4-diyl)diacetamide	212.0778	1.14		
$C_7H_8ClN_5O_2$	CDDT	N,N'-(6-Chloro-1,3,5-triazine-2,4-diyl)diacetamide	230.0440	1.45		

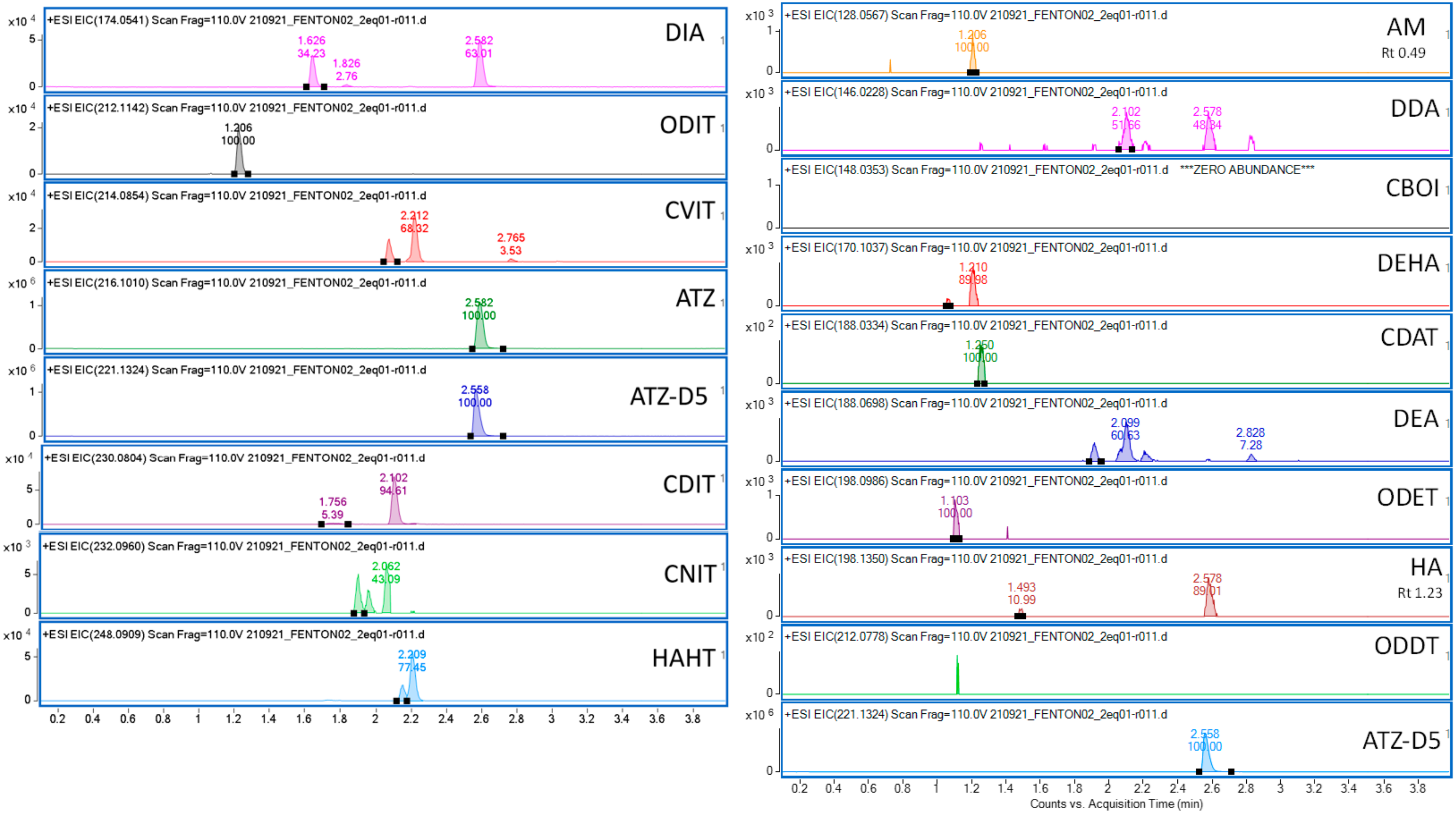

Figure S1. Extracted ion chromatogram LC-ESI+ - HRMS during the degradation of atrazine in automatic sampling during the experiment of 2 eq. Fenton reagents (m/z ± 20 ppm).

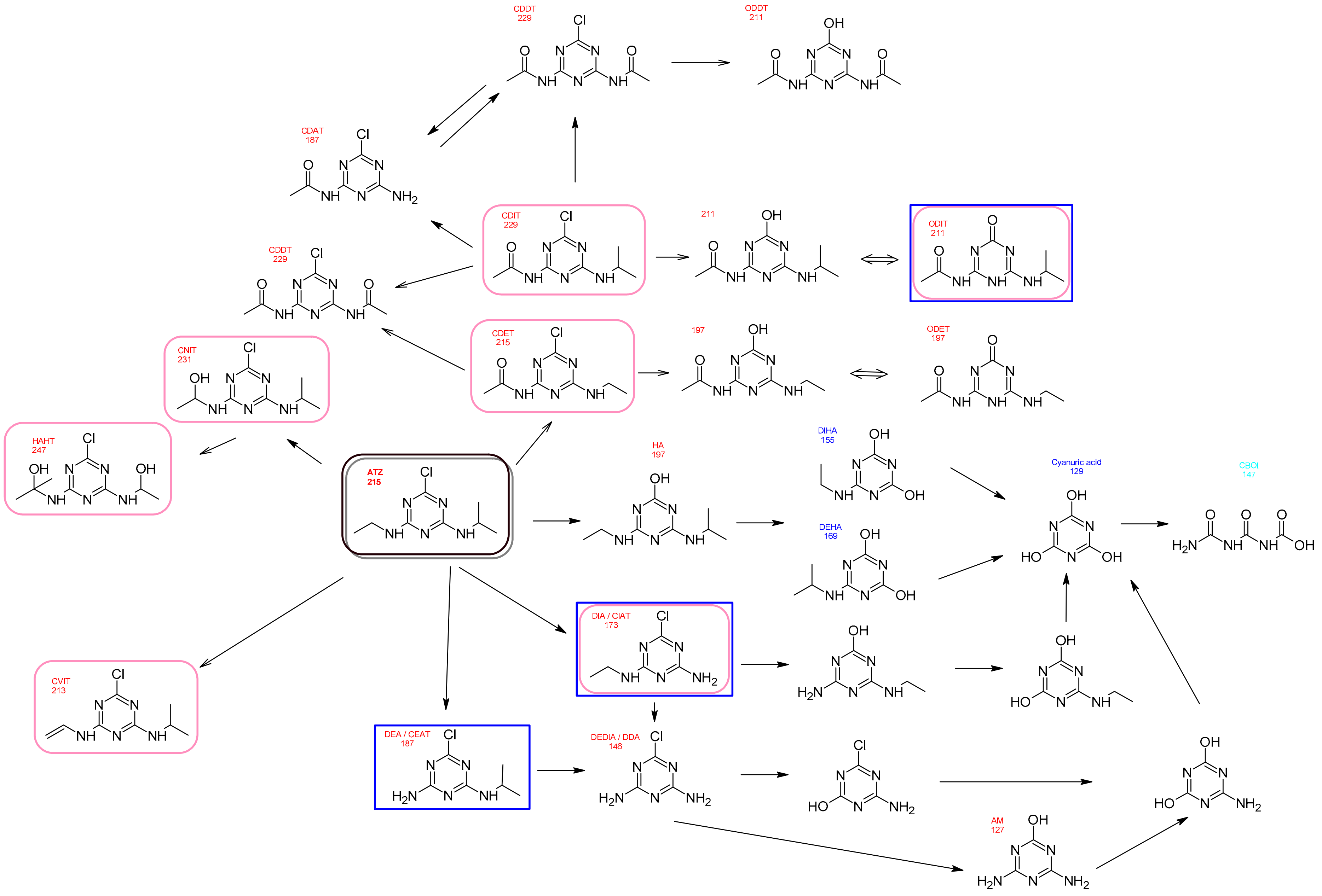

Figure S2. Proposed atrazine degradation pathways scheme. The red and blue boxes respectively indicate the main products of Fenton oxidation (2 eq. Fenton reagents) and ultrasound treatment (US 50 kHz + Fe^{2+}) after 50 min, according to the data from table SI.1.

Table S2. List of metabolites targets.

Description	CAS number	Molecular formula	Chemical structure	Log P	Monoisotopic mass	[M+H]$^+$	Reference
Atrazine (ATZ)	1912-24-9	$C_8H_{14}ClN_5$	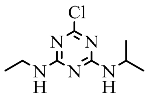	2.63	215.0938	216.1011	1, 2, 3, 4, 5, 6, 7, 8, 9, 10, 11, 12, 13, 14, 15, 16, 17, 18, 19, 20, 21, 22, 23, 24, 25, 26, 27, 28, 29, 30, 31, 32, 33, 34, 35, 36, 37, 38, 39, 40, 41, 42, 43, 44, 45, 46, 47, 48, 49, 50, 51, 52
Deethylatrazine (DEA)	6190-65-4	$C_6H_{10}ClN_5$	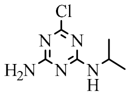	1.20	187.0625	188.0698	1, 2, 3, 4, 5, 7, 9, 10, 11, 12, 14, 15, 16, 17, 18, 20, 21, 22, 23, 24, 25, 26, 27, 30, 31, 32, 33, 35, 36, 37, 38, 40, 41, 42, 43, 44, 45, 46, 47, 48, 49, 50, 52
Deisopropylatrazine (DIA)	1007-28-9	$C_5H_8ClN_5$	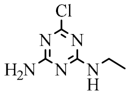	1.2	173.0468	174.0542	1, 2, 3, 4, 5, 7, 9, 10, 11, 12, 14, 15, 16, 17, 18, 20, 21, 22, 24, 25, 26, 27, 30, 31, 32, 33, 35, 36, 37, 38, 40, 41, 42, 43, 44, 45, 46, 47, 49, 52
Hydroxyatrazine (HA)	2163-68-0	$C_8H_{15}N_5O$	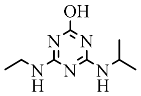	-0.87	197.1277	198.1350	2, 3, 4, 5, 6, 7, 8, 10, 11, 13, 14, 15, 17, 18, 19, 23, 24, 25, 26, 27, 28, 29, 30, 31, 32, 33, 34, 36, 37, 38, 39, 41, 43, 44, 45, 47, 51, 52

Description	CAS number	Molecular formula	Chemical structure	Log P	Monoisotopic mass	$[M+H]^+$	Reference
Atrazine amide (CDIT)	83364-15-2	$C_8H_{12}ClN_5O$	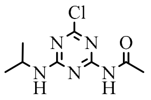	2.063	229.0730	230.0804	1, 9, 11, 12, 15, 16, 17, 21, 22, 25, 26, 27, 30, 32, 33, 34, 35, 36, 39, 43, 48, 50, 52
Simazine amide (CDET)	142179-76-8	$C_7H_{10}ClN_5O$	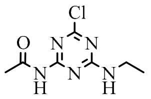	1.409	215.0574	216.0647	17, 25, 39
1-({4-chloro-6-[(propan-2-yl)amino]-1,3,5-triazin-2-yl}amino)ethan-1-ol (CNIT)	169523-79-9	C8H14ClN5O	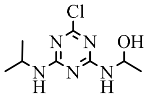	1.892	231.0887	232.0960	7, 9, 11, 15, 17, 21, 26, 30, 32, 33, 35, 36, 39, 43, 47, 50, 52
Didealkylatrazine (DDA)	3397-62-4	$C_3H_4ClN_5$	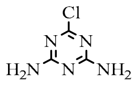	-1.05	145.0155	146.0228	1, 2, 3, 4, 5, 7, 9, 11, 12, 13, 14, 15, 17, 18, 20, 21, 22, 23, 24, 25, 26, 27, 28, 30, 31, 33, 34, 36, 38, 39, 40, 41, 42, 43, 44, 45, 46, 48, 49, 52
Deethylhydroxyatrazine (DEHA)	19988-24-0	$C_6H_{11}N_5O$	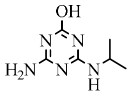	-1.46	169.0964	170.1037	3, 5, 7, 8, 11, 12, 15, 17, 24, 25, 26, 30, 33, 34, 36, 37, 38, 39, 40, 43, 45, 48, 49, 50, 52

Description	CAS number	Molecular formula	Chemical structure	Log P	Monoisotopic mass	$[M+H]^+$	Reference
Deisopropylhydroxyatrazine (DIHA)	7313-54-4	$C_5H_9N_5O$	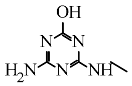	-1.403	155.0807	156.0880	3, 5, 7, 8, 18, 19, 24, 25, 34, 36, 38, 39, 43, 44, 45, 49
Hydroxyatrazine amide (ODIT)	144868-61-1	$C_8H_{13}N_5O_2$	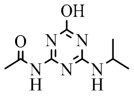	-1.02	211.1069	212.1142	1, 7, 11, 12, 16, 17, 21, 26, 30, 31, 33, 34, 35, 36, 39, 43, 48, 50, 52
Deisopropylatrazine amide (CDAT)	115339-34-9	$C_5H_6ClN_5O$	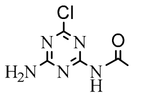	-1.1	187.0261	188.0334	1, 15, 17, 25, 30, 39
N,N'-(6-Chloro-1,3,5-triazine-2,4-diyl)diacetamide (CDDT)	143593-21-9	C7H8ClN5O2	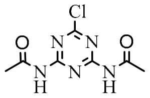	-0.14	229.0367	230.0440	2, 17, 25, 39
N-[6-(Ethylamino)-4-oxo-1,4-dihydro-1,3,5-triazin-2-yl]acetamide (ODET)	860473-84-3	C7H11N5O2	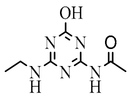	-1.13	197.0913	198.0986	6, 17, 27, 34, 39, 51

Description	CAS number	Molecular formula	Chemical structure	Log P	Monoisotopic mass	$[M+H]^+$	Reference
2-({4-chloro-6-[(1-hydroxyethyl)amino]-1,3,5-triazin-2-yl}amino)propan-2-ol (HAHT)	1242141-71-4	C8H14ClN5O2	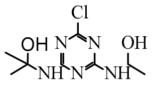	2.365	247.0836	248.0909	7, 39
6-chloro-N2-ethenyl-N4-(propan-2-yl)-1,3,5-triazine-2,4-diamine (CVIT)	169523-77-7	C8H12ClN5	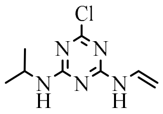	2.312	213.0781	214.0854	11, 17, 31, 43, 50
Ammeline (AM)	645-92-1	$C_3H_5N_5O$	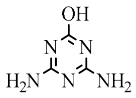	-1.281	127.0494	128.0567	3, 5, 6, 7, 8, 11, 12, 14, 15, 17, 19, 21, 25, 26, 27, 28, 30, 32, 33, 34, 35, 36, 39, 40, 43, 44, 45, 48, 49, 50, 52
N,N'-(6-hydroxy-1,3,5-triazine-2,4-diyl)diacetamide (ODDT)	914486-10-5	C7H9N5O3	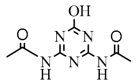	-1.057	211.0705	212.0779	17, 27
1-carboxybiuret (CBOI)	1329090-18-7	C3H5N3O4	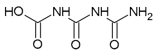	-1.976	147.0280	148.0353	

Description	CAS number	Molecular formula	Chemical structure	Log P	Monoisotopic mass	$[M+H]^+$	Reference
Atrazine-D5	163165-75-1	C8H9D5ClN5	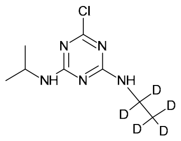	2.63	220.1252	221.1325	

Reference :

1. Acero, J.L., K. Stemmler, and U. von Gunten, Degradation Kinetics of Atrazine and Its Degradation Products with Ozone and OH Radicals: A Predictive Tool for Drinking Water Treatment. Environmental Science & Technology, 2000. 34(4): p. 591-597.

2. Aggelopoulos, C.A., D. Tataraki, and G. Rassias, Degradation of atrazine in soil by dielectric barrier discharge plasma – Potential singlet oxygen mediation. Chemical Engineering Journal, 2018. 347: p. 682-694.

3. Balci, B., et al., Degradation of atrazine in aqueous medium by electrocatalytically generated hydroxyl radicals. A kinetic and mechanistic study. Water Research, 2009. 43(7): p. 1924-1934.

4. Basfar, A.A., et al., Radiolytic degradation of atrazine aqueous solution containing humic substances. Ecotoxicology and Environmental Safety, 2009. 72(3): p. 948-953.

5. Bianchi, C.L., et al., Mechanism and efficiency of atrazine degradation under combined oxidation processes. Applied Catalysis B: Environmental, 2006. 64(1): p. 131-138.

6. Chen, S., et al., Co/Sm-modified Ti/PbO2 anode for atrazine degradation: Effective electrocatalytic performance and degradation mechanism. Chemosphere, 2021. 268: p. 128799.

7. Deng, S., et al., Mechanochemically synthesized S-ZVIbm composites for the activation of persulfate in the pH-independent degradation of atrazine: Effects of sulfur dose and ball-milling conditions. Chemical Engineering Journal, 2021. 423: p. 129789.

8. Fernández-Domene, R.M., et al., Elimination of pesticide atrazine by photoelectrocatalysis using a photoanode based on WO3 nanosheets. Chemical Engineering Journal, 2018. 350: p. 1114-1124.

9. Granados-Oliveros, G., et al., Degradation of atrazine using metalloporphyrins supported on TiO2 under visible light irradiation. Applied Catalysis B: Environmental, 2009. 89(3): p. 448-454.

10. Hu, E. and H. Cheng, Catalytic effect of transition metals on microwave-induced degradation of atrazine in mineral micropores. Water Research, 2014. 57: p. 8-19.

11. Huang, Y., et al., Degradation of atrazine by ZnxCu1−xFe2O4 nanomaterial-catalyzed sulfite under UV–vis light irradiation: Green strategy to generate SO4−. Applied Catalysis B: Environmental, 2018. 221: p. 380-392.

12. Jiang, Q., et al., Graphene-like carbon sheet-supported nZVI for efficient atrazine oxidation degradation by persulfate activation. Chemical Engineering Journal, 2021. 403: p. 126309.

13. Khan, J.A., N.S. Shah, and H.M. Khan, Decomposition of atrazine by ionizing radiation: Kinetics, degradation pathways and influence of radical scavengers. Separation and Purification Technology, 2015. 156: p. 140-147.

14. Khan, J.A., et al., Role of eaq−, OH and H in radiolytic degradation of atrazine: A kinetic and mechanistic approach. Journal of Hazardous Materials, 2015. 288: p. 147-157.

15. Li, C., et al., Highly efficient activation of peroxymonosulfate by natural negatively-charged kaolinite with abundant hydroxyl groups for the degradation of atrazine. Applied Catalysis B: Environmental, 2019. 247: p. 10-23.

16. Li, G., et al., Intrinsic mechanisms of calcium sulfite activation by siderite for atrazine degradation. Chemical Engineering Journal, 2021. 426: p. 131917.

17. Liu, B., et al., Activation of peroxymonosulfate by cobalt-impregnated biochar for atrazine degradation: The pivotal roles of persistent free radicals and ecotoxicity assessment. Journal of Hazardous Materials, 2020. 398: p. 122768.

18. Lu, Y.C., et al., Genome-wide identification of DNA methylation provides insights into the association of gene expression in rice exposed to pesticide atrazine. Scientific Reports, 2016. 6(1): p. 18985.

19. Mahlalela, L.C., et al., Photocatalytic degradation of atrazine in aqueous solution using hyperbranched polyethyleneimine templated morphologies of BiVO4 fused with Bi2O3. Journal of Environmental Chemical Engineering, 2020. 8(5): p. 104215.

20. McBeath, S.T. and N.J.D. Graham, In-situ electrochemical generation of permanganate for the treatment of atrazine. Separation and Purification Technology, 2021. 260: p. 118252.

21. Peng, J., et al., Degradation of atrazine by persulfate activation with copper sulfide (CuS): Kinetics study, degradation pathways and mechanism. Chemical Engineering Journal, 2018. 354: p. 740-752.

22. Petrier, C., B. David, and S. Laguian, Ultrasonic degradation at 20 kHz and 500 kHz of atrazine and pentachlorophenol in aqueous solution: Preliminary results. Chemosphere, 1996. 32(9): p. 1709-1718.

23. Qu, M., et al., Phytoextraction and biodegradation of atrazine by Myriophyllum spicatum and evaluation of bacterial communities involved in atrazine degradation in lake sediment. Chemosphere, 2018. 209: p. 439-448.

24. Saltmiras, D.A. and A.T. Lemley, Atrazine degradation by anodic Fenton treatment. Water Research, 2002. 36(20): p. 5113-5119.

25. Saylor, G.L., C. Zhao, and M.J. Kupferle, Synergistic enhancement of oxidative degradation of atrazine using combined electrolysis and ozonation. Journal of Water Process Engineering, 2018. 21: p. 154-162.

26. Sun, X., H. Qi, and Z. Sun, Bifunctional nickel foam composite cathode co-modified with CoFe@NC and CNTs for electrocatalytic degradation of atrazine over wide pH range. Chemosphere, 2022. 286: p. 131972.

27. Ta, N., et al., Degradation of atrazine by microwave-assisted electrodeless discharge mercury lamp in aqueous solution. Journal of Hazardous Materials, 2006. 138(1): p. 187-194.

28. Teng, X., et al., Effective degradation of atrazine in wastewater by three-dimensional electrochemical system using fly ash-red mud particle electrode: Mechanism and pathway. Separation and Purification Technology, 2021. 267: p. 118661.

29. Udiković-Kolić, N., C. Scott, and F. Martin-Laurent, Evolution of atrazine-degrading capabilities in the environment. Applied Microbiology and Biotechnology, 2012. 96(5): p. 1175-1189.

30. Wang, G., et al., Enhanced degradation of atrazine by nanoscale LaFe1-xCuxO3-δ perovskite activated peroxymonosulfate: Performance and mechanism. Science of The Total Environment, 2019. 673: p. 565-575.

31. Wang, Q., et al., Degradation of aqueous atrazine using persulfate activated by electrochemical plasma coupling with microbubbles: removal mechanisms and potential applications. Journal of Hazardous Materials, 2021. 403: p. 124087.

32. Wang, T., et al., Electrochemical degradation of atrazine by BDD anode: Evidence from compound-specific stable isotope analysis and DFT simulations. Chemosphere, 2021. 273: p. 129754.

33. Wang, W.-K., et al., Photocatalytic degradation of atrazine by boron-doped TiO2 with a tunable rutile/anatase ratio. Applied Catalysis B: Environmental, 2016. 195: p. 69-76.

34. Wang, X., et al., Pyrite enables persulfate activation for efficient atrazine degradation. Chemosphere, 2020. 244: p. 125568.

35. Wu, S., et al., Insights into atrazine degradation by persulfate activation using composite of nanoscale zero-valent iron and graphene: Performances and mechanisms. Chemical Engineering Journal, 2018. 341: p. 126-136.

36. Wu, S., et al., Performances and mechanisms of efficient degradation of atrazine using peroxymonosulfate and ferrate as oxidants. Chemical Engineering Journal, 2018. 353: p. 533-541.

37. Xie, S., et al., Highly efficient photoelectrochemical removal of atrazine and the mechanism investigation: Bias potential effect and reactive species. Journal of Hazardous Materials, 2021. 415: p. 125681.

38. Xu, G., et al., Electron beam induced degradation of atrazine in aqueous solution. Chemical Engineering Journal, 2015. 275: p. 374-380.

39. Xu, L.J., W. Chu, and N. Graham, Atrazine degradation using chemical-free process of USUV: Analysis of the micro-heterogeneous environments and the degradation mechanisms. Journal of Hazardous Materials, 2014. 275: p. 166-174.

40. Xu, X., et al., Atrazine degradation using Fe3O4-sepiolite catalyzed persulfate: Reactivity, mechanism and stability. Journal of Hazardous Materials, 2019. 377: p. 62-69.

41. Yang, J., et al., Study on enhanced degradation of atrazine by ozonation in the presence of hydroxylamine. Journal of Hazardous Materials, 2016. 316: p. 110-121.

42. Yang, Y., et al., Degradation and transformation of atrazine under catalyzed ozonation process with TiO2 as catalyst. Journal of Hazardous Materials, 2014. 279: p. 444-451.

43. Ye, G., et al., Three-dimensional Co/Ni bimetallic organic frameworks for high-efficient catalytic ozonation of atrazine: Mechanism, effect parameters, and degradation pathways analysis. Chemosphere, 2020. 253: p. 126767.

44. Yola, M.L., T. Eren, and N. Atar, A novel efficient photocatalyst based on TiO2 nanoparticles involved boron enrichment waste for photocatalytic degradation of atrazine. Chemical Engineering Journal, 2014. 250: p. 288-294.

45. Yu, T., et al., A bio-functions integration microcosm: Self-immobilized biochar-pellets combined with two strains of bacteria to remove atrazine in water and mechanisms. Journal of Hazardous Materials, 2020. 384: p. 121326.

46. Yuan, X., et al., Enhanced ozonation degradation of atrazine in the presence of nano-ZnO: Performance, kinetics and effects. Journal of Environmental Sciences, 2017. 61: p. 3-13.

47. Zhang, J.J., Y.C. Lu, and H. Yang, Chemical Modification and Degradation of Atrazine in Medicago sativa through Multiple Pathways. Journal of Agricultural and Food Chemistry, 2014. 62(40): p. 9657-9668.

48. Zhang, R., et al., Efficient degradation of atrazine by LaCoO3/Al2O3 catalyzed peroxymonosulfate: Performance, degradation intermediates and mechanism. Chemical Engineering Journal, 2019. 372: p. 796-808.

49. Zhang, Y., et al., The role of dissolved oxygen in the Ta(O)N-driven visible Fenton-like degradation of atrazine. Journal of Environmental Chemical Engineering, 2014. 2(3): p. 1691-1698.

50. Zhang, Y., et al., One-step synthesis of biochar supported nZVI composites for highly efficient activating persulfate to oxidatively degrade atrazine. Chemical Engineering Journal, 2021. 420: p. 129868.

51. Zhanqi, G., et al., Microwave assisted rapid and complete degradation of atrazine using TiO2 nanotube photocatalyst suspensions. Journal of Hazardous Materials, 2007. 145(3): p. 424-430.

52. Zhu, S., et al., Heterogeneous catalysis of ozone using ordered mesoporous Fe3O4 for degradation of atrazine. Chemical Engineering Journal, 2017. 328: p. 527-535.
